# Cluj hosts the first European Summer School of Quantitative Electroencephalography (QEEG) – Blended Intensive Program within the European University of Brain and Technology (Neurotech^EU^)

**DOI:** 10.25122/jml-2022-1025

**Published:** 2022-09

**Authors:** Livia Livinț Popa, Hanna-Maria DragoÈ™, Victor Dăbală, Diana Chertic, Irina Vlad, Ștefan Strilciuc, Dafin-Fior Mureșanu

**Affiliations:** 1 Department of Neurosciences, Iuliu Hațieganu University of Medicine and Pharmacy, Cluj-Napoca, Romania; 2 RoNeuro Institute for Neurological Research and Diagnostic, Cluj-Napoca, Romania

## INTRODUCTION

QEEG is a modern electroencephalography (EEG) analysis that records digital EEG signals, which are processed, transformed, and analyzed using complex mathematical algorithms. When Hans Berger recorded the first EEG of a human brain in 1924, his observations were limited to the time domain. Still, he suggested that frequency analysis would improve the interpretation of EEG signals in the future. QEEG has brought new EEG signal feature extraction techniques: analysis of specific frequency band and signal complexity, analysis of connectivity, and network analysis. The role of QEEG is not necessarily to pinpoint an immediate diagnosis but to provide additional insight in conjunction with other diagnostic assessments for obtaining a precise result and disease severity stage or specific treatment response evaluation. The clinical application of QEEG is extensive, including neuropsychiatric disorders, epilepsy, stroke, dementia, traumatic brain injury, and many others.

Neurotechnology establishes strategic connections between various disciplines, from neuroscience and medicine to engineering and artificial intelligence, tackling the more profound understanding of fundamental brain principles and enhancing the advancement of new technological methods in experimental and clinical settings.

Neurotech^EU^, the European University of Brain and Technology, brings together eight leading European universities, partner research institutions, companies, societal stakeholders, cities, and (non) governmental organizations to build a trans-European network of excellence in brain research and technologies to increase the competitiveness of European education, research, economy, and society. In this context, the University of Medicine and Pharmacy Iuliu Hațieganu (UMFIH), through the Erasmus+ department, developed the first Blended Intensive Program (BIP) within the Neurotech^EU^ Alliance, organizing the QEEG Summer School (QEEGSS). BIP aims to open more opportunities for learners to participate in blended mobilities, supporting the development of transnational and transdisciplinary curricula and innovative teaching methods such as research-based learning and challenge-based approaches.

The first edition of the QEEGSS educational program was conducted in Cluj-Napoca, Romania, between July 11^th^ and 15^th^, 2022, to promote a basic understanding of QEEG analysis and its applications in a multidisciplinary environment. This innovative event was the first BIP of the Neurotech^EU^ alliance, developed with its academic partners: UMFIH through Erasmus+ department and RoNeuro Institute for Neurological Research and Diagnostic.

The five-day training followed a simple-to-complex approach, from the source and principles of EEG to the most complex types of QEEG analysis. It provided early-stage researchers with a basic yet modern understanding of where QEEG has arrived today and potential avenues for its development. QEEGSS brought together junior researchers with a common interest in QEEG and distinctive backgrounds (*e.g*., medical school students, psychologists, neuroscience or PhD students) from six European countries (Germany, Bulgaria, the Netherlands, Slovenia, Hungary, and Romania), facilitating multidisciplinary interaction and exchange of skills.

The scientific part of the QEEGSS program consisted of theoretical courses and hands-on activities. The theoretical courses aimed to provide an overarching view of the principles, technical details, basic interpretation, and clinical applications of EEG in defining and understanding the complexity of analysis and the role of QEEG in research and clinical practice. Moreover, drawing inspiration from the general outlines of neural communication concepts, the lectures presented the mathematical notions and experimental settings of the three domains of analysis in QEEG:

1. Frequency domain;
2. Time domain;
3. Frequency-time domain.

Central importance for QEESS was to familiarize the participants with several tools of QEEG analysis such as EEGLAB and Brain Vision Analyzer throughout the hands-on sessions, enhancing team-working activities and multidisciplinary approach. The lectures and hands-on sessions showcased the principles, the types of analysis, and applications of QEEG from different perspectives depending on research and clinical backgrounds, all highlighting the importance of performing and interpreting the QEEG after conventional EEG was priorly conducted.

**Figure 1:**
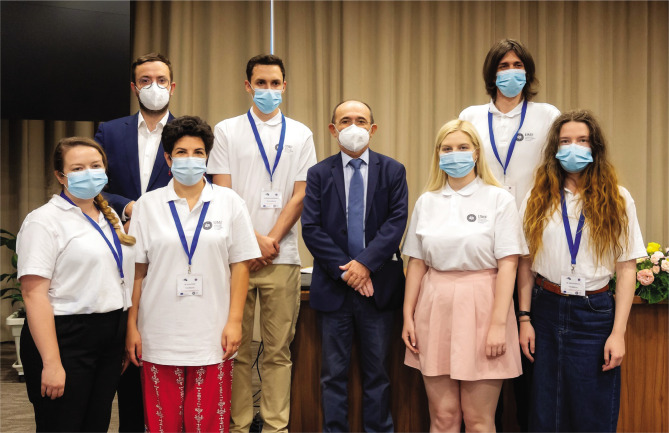
Speakers at the QEEG Summer School.

The QEEG Lab team of RoNeuro conducted research activities on spectral and connectivity analysis of QEEG and its applications in different neuropsychiatric disorders for at least four years under the guidance of Prof. Dafin Mureșanu, president of the European Federation of Neurorehabilitation Societies (EFNR) and the Society for the Study of Neuroprotection and Neuroplasticity (SSNN) (Figure 1).

This educational and practical event was set up to unfold over 5 days in two locations: UMFIH on the first two days and RoNeuro on the following days for the hands-on sessions. Furthermore, as part of the BIP recommendations, the final lecture was developed as a virtual course with innovative interactive resources. A total of 23 delegates from different countries participated in the intensive program: Germany – Bonn University (6 delegates), Bulgaria – Medical University of Sofia (4 delegates), the Netherlands – Radboud University (3 delegates), Slovenia – Maribor University (2 delegates), Hungary – Debrecen University (1 delegate), Romania – UMFIH (6 delegates), Carol Davila University of Medicine and Pharmacy, București (1 delegate).

Participants were given the opportunity to share their knowledge and experience on conventional EEG, discuss and question basic concepts, and analysis of QEEG. Ultimately, the delegates were divided into five teams, and the goal of each team was to develop a project idea that involved the QEEG assessment of subjects with specific pathology. Then, being provided with a potential methodological approach, they were invited to build up a research proposal and an elevator pitch as they were applying for a grant, convincing the evaluation board that their ideas were relevant and feasible.

All participants were early-stage researchers with distinctive backgrounds from medical sciences up to psychology, with an interest in the QEEG assessment of neuropsychiatric physiology and pathology.

## EVENT SUMMARY

Prof. Dafin Fior Mureşanu opened the QEEGSS with an overview of the new paradigms in neurophysiology research and the importance of extending the knowledge about neuroscience inside an ever-increasing technology in this field.

After the introduction, the participants were divided into five teams and encouraged to propose a research hypothesis aligned with their shared interests, which could be tested through QEEG. Each team came up with creative project proposals, such as:

- The effects of antidepressants on sleep architecture in offspring rodents with a history of prenatal stress;
- The benefits of mindfulness meditation in young ADHD subjects;
- The effects of imagination of motor action on mu waves.

**Figure 2:**
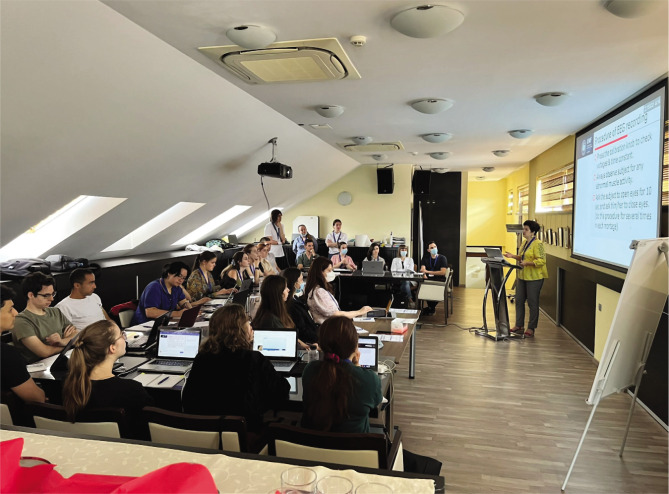
Plenary lecture on EEG basic concepts.

In the second part of the day, Dr. Livia Popa lectured on the fundamentals of EEG, describing the sources of the electric scalp activity and recording principles (electrode placement, montages, filters, calibration) (Figure 2). Through interactive methods, the participants discovered the clinical applications of EEG and different types of pathological activity. As the millisecond complex dynamics of the human cortex cannot be quantified on conventional EEG, QEEG is gaining more and more ground in the basic and medical sciences. The next point of her presentation was an introduction to signal transformation and data analysis using linear and non-linear QEEG methods.

The following presentations focused on spectral analysis, time-frequency decomposition, and various connectivity analyses. First, Dr. Victor Dăbală presented how a Fourier transformation (FT) works, emphasizing the Fast Fourier Transform (FFT) and the convolution theorem. Next, Dr. Diana Chertic introduced the most used methods for obtaining time-resolved frequency representations of EEG data and for assessing the connectivity in the time domain. Finally, Dr. Hanna Dragoș covered the basics of several connectivity methods such as coherence, phase-locking value, and the Granger causality. By being asked to fixate on a central flashing dot while ignoring the dots existing anywhere else in the visual field, the participants were introduced to the concepts of selective attention and motion-induced blindness as changes in communication structure between brain areas. QEEG applications in daily neurological practice were highlighted through clinical cases that entertained the participants in debates on differential diagnoses from neurodevelopmental to neurodegenerative disorders.

The third day occurred at the RoNeuro Institute for Neurological Research and Diagnostic. The institute has a multidisciplinary clinical activity and a research branch driving innovation in neuroscience across sectors. The day started with Dr. Livia Popa offering an interactive and comprehensive presentation about EEG patterns, types of activity, and artifacts. The participants were also instructed on the features of EEG in neurological disorders, comprising a full dive into the complex concept of EEG.

Dr. Ștefan Strilciuc then coordinated a hands-on experience targeting the Independent Component Analysis (ICA) technique, used for two fundamental QEEG steps: cleansing the data prior to the analysis and identifying the brain sources of specific signal patterns.

The hands-on session continued on the fourth day, when the participants could record the EEG of a randomly assigned participant. Afterwards, they were instructed on the preprocessing and analysis of the recorded signal, both activities led by Dr. Livia Popa. Using the EEGLAB software, the time series data was first referenced, filtered, cleared from artifacts and bad segments, and analyzed using FFT and time-frequency decomposition. Some source reconstruction techniques were also employed, and the participants were introduced to the forward and inverse problems.

The last day of QEEGSS started with an online lecture, which offered a basic overview of several technologies that can be used in conjunction with QEEG, covering their development, functions, mechanisms, and research applications. Therefore, concepts like transcranial magnetic stimulation (TMS), functional magnetic resonance imaging (fMRI), functional near-infrared spectroscopy (fNIRS), eye tracking (ET), magnetoencephalography (MEG), transcranial direct-curent stimulation (tDCS) and transcranial alternating current stimulation (tACS) were developed through innovative interactive virtual resources by Dr. Irina Vlad.

QEEG participants had the opportunity to enrich their local experiences by attending various leisure and cultural activities, such as familiarizing with the Romanian culture and language, exploring the Turda Salt Mine (recently considered for UNESCO heritage inclusion), sharing a multicultural dinner, experiencing a deep dive into the history and architectural landmarks of Cluj-Napoca through a guided visit to the city's historical center, and, lastly, exploring the Alexandru Borza Botanical Garden.

**Figure 3:**
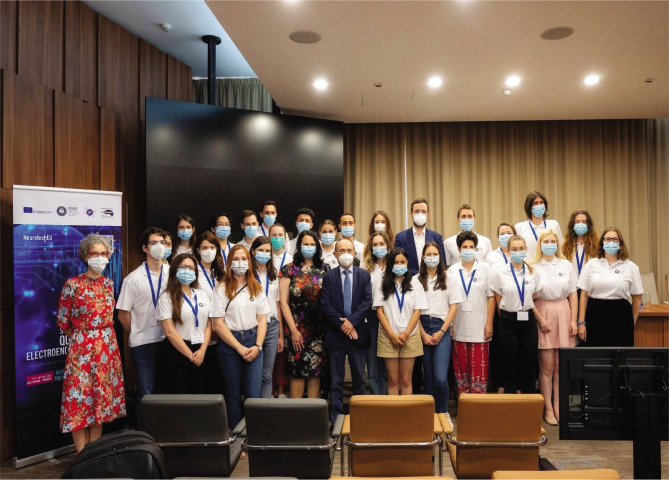
QEEG Summer School – Closing Ceremony.

The QEEGSS lectors and participants (Figure 3) contributed, throughout the five days of the hybrid educational event, to implementing an insightful and innovative multidisciplinary program that outlined the utmost benefits of knowledge dissemination, shared experiences, and multimodal approaches.

## CHALLENGES AND OPPORTUNITIES

The first edition of QEEGSS marked the beginning of BIP educational events within the alliance of Neurotech^EU^. As quantitative electroencephalography is becoming a reliable tool for experts in the interconnected fields of neurosciences and biotechnology, the event cleared a possible pathway regarding the interest of future generations in exploring the boundaries of their field of expertise.

Considering the critical significance of international collaboration and the continuous need for multidimensional and multidisciplinary approaches for neurotechnology applications in research settings, the QEEGSS offered a peak into QEEG and its potential to drive developments in clinical neuroscience and beyond.

